# Ultrasonographic diagnosis of pure inversion of Meckel’s diverticulum without obstruction in a child: a case report and literature review

**DOI:** 10.3389/fmed.2026.1770539

**Published:** 2026-03-13

**Authors:** Shasha Chen, Tingting Shen, Yunfang Zhu, Hongxia Yuan, Rong Tian, Xingxing Duan

**Affiliations:** Changsha Hospital for Maternal and Child Health Care, Changsha, Hunan, China

**Keywords:** diagnostic value, high-frequency ultrasonography, inversion, Meckel’s diverticulum, target sign

## Abstract

**Introduction:**

Meckel’s diverticulum (MD) is the most common congenital malformation of the gastrointestinal tract. Most cases are asymptomatic, while a minority may develop complications such as hematochezia, diverticulitis, and intestinal obstruction. When a Meckel’s diverticulum inverts into the lumen of the ileum rather than protruding outward from the intestinal wall, it is referred to as an inverted Meckel’s diverticulum, which is a very rare condition. Although inverted MD frequently presents with intestinal obstruction, cases of pure inversion without obstruction are exceptionally rare. However, even in the absence of obstruction, such cases may still manifest with non-specific symptoms, posing a significant diagnostic challenge. This report describes a case of complete MD inversion in a child that was accurately diagnosed by high-frequency ultrasonography (HFUS), with preoperative ultrasound findings consistent with intraoperative observations.

**Case description:**

A 5-year-old child presented with recurrent abdominal pain and diarrhea for 4 months, having been evaluated at multiple hospitals without a definitive diagnosis. Ultrasound examination at our institution revealed a Meckel’s diverticulum in the terminal ileum; however, instead of protruding outward, the diverticulum was inverted into the intestinal lumen. This invagination into the lumen explained the patient’s clinical manifestations of abdominal pain and diarrhea. Ultrasound diagnosis was MD inversion. After preoperative evaluation, the patient underwent transumbilical single-port laparoscopic partial small bowel resection and anastomosis, which intraoperatively confirmed the diagnosis of inverted MD.

**Conclusion:**

This case demonstrates that high-frequency ultrasonography can accurately identify an inverted Meckel’s diverticulum by revealing the characteristic morphological changes of the diverticulum within the ileal lumen. This imaging modality proved valuable in establishing the correct preoperative diagnosis and guiding timely surgical intervention.

## Introduction

Meckel’s diverticulum (MD), a remnant of the incompletely obliterated omphalomesenteric duct, is typically located on the anti-mesenteric border of the distal ileum, projecting outward from the intestinal lumen (1, 2). As the most common congenital gastrointestinal malformation, MD has a prevalence of approximately 2% in the population (3). While most cases are asymptomatic, some may lead to digestive complications such as hematochezia, diverticulitis, and intestinal obstruction. The inversion of MD into the intestinal lumen, termed inverted MD, is rare and poses a preoperative diagnostic challenge (4). Pure inversion of a Meckel’s diverticulum without obstruction is an exceptionally rare phenomenon. Importantly, even in the absence of obstruction, affected patients may still experience non-specific symptoms, rendering preoperative identification of this entity a significant diagnostic challenge. In this article, we report a case of complete MD inversion in a child that high-frequency ultrasonography (HFUS) accurately diagnosed. This case demonstrates that high-frequency ultrasonography can accurately identify an inverted Meckel’s diverticulum by revealing the characteristic morphological changes of the diverticulum within the ileal lumen. This imaging modality proved valuable in establishing the correct preoperative diagnosis and guiding timely surgical intervention.

## Case presentation

**Figure 1:**
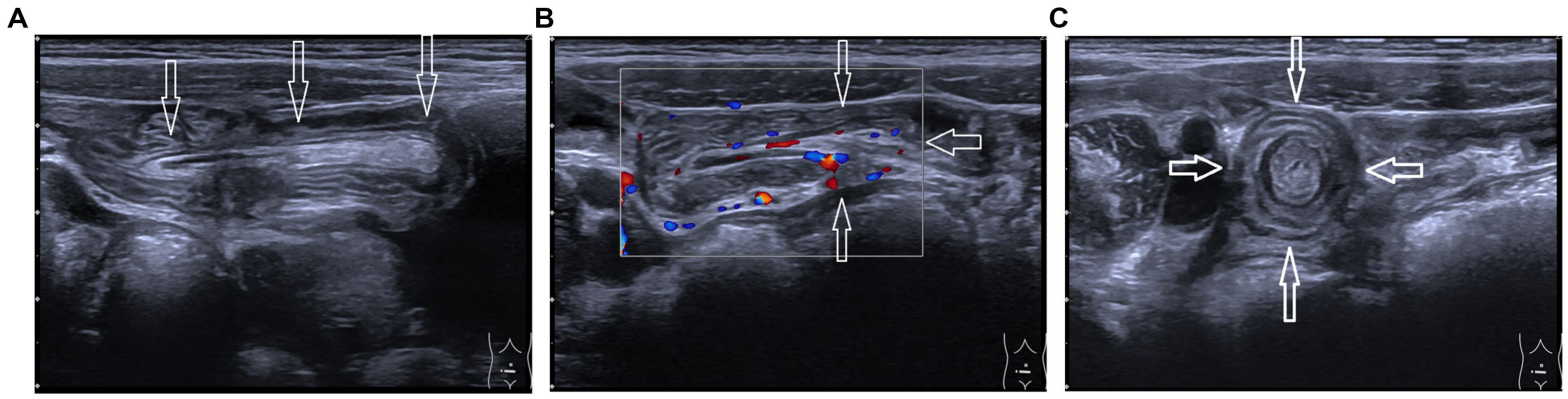
High-frequency ultrasonographic images of inverted Meckel’s diverticulum. **(A)** White arrow indicates the “glove-finger”-shaped protrusion into the ileal lumen. **(B)** The white arrow shows the hyperechoic mesentery and tubular anechoic mesenteric vessels extending outward and continuing with the adjacent mesentery. **(C)** Transverse section displays the “target sign” with a hyperechoic center (arrow).

**Figure 2:**
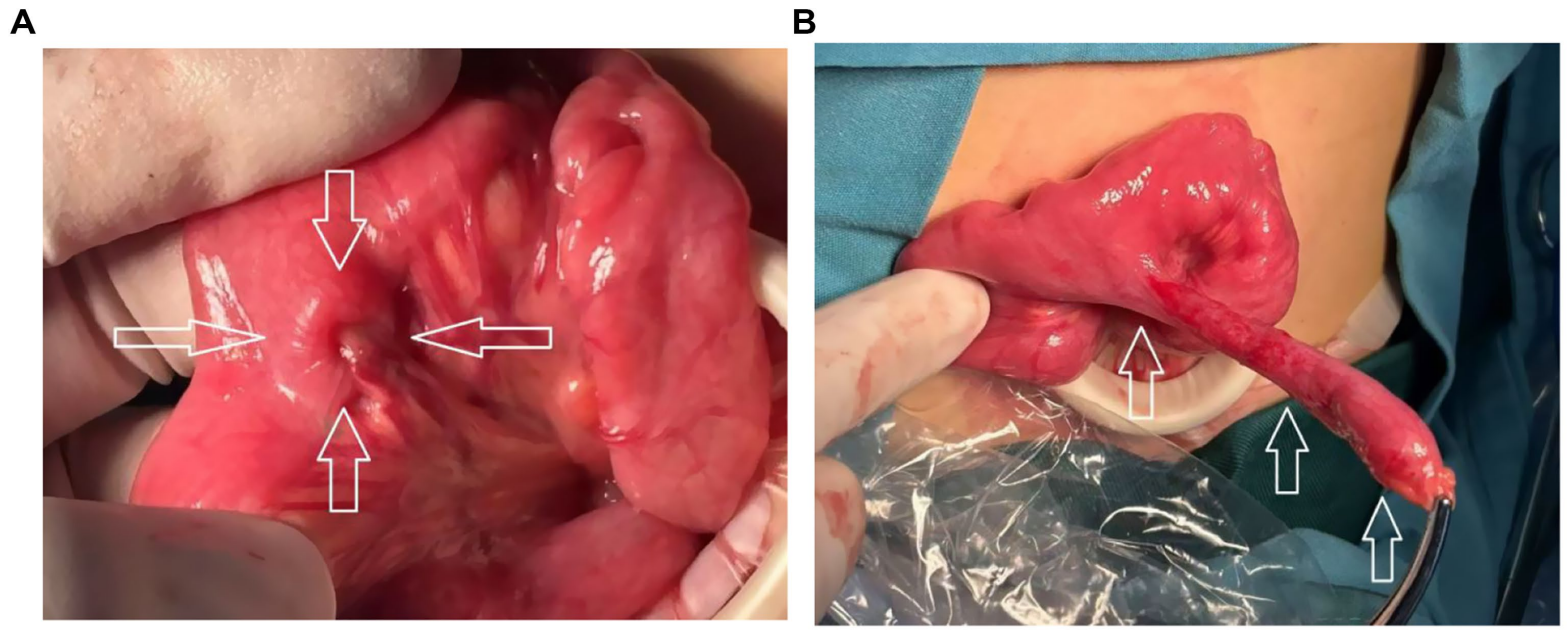
Intraoperative images of inverted Meckel’s diverticulum. **(A)** Meckel’s diverticulum (MD) inverted into the ileum, showing an “umbilical”-like appearance (arrow). **(B)** The MD after reduction, demonstrating a “glove-finger”-shaped appearance (arrow).

**Figure 3:**
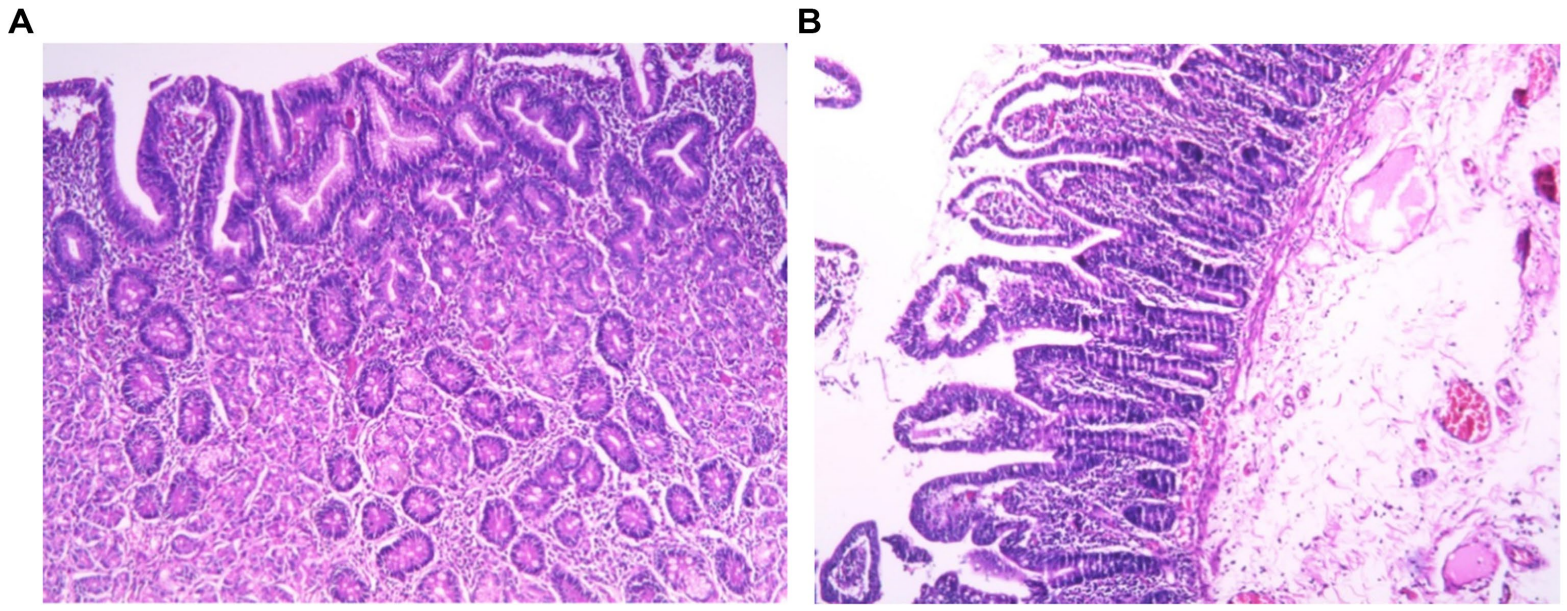
Histopathological sections of Meckel’s diverticulum. **(A)** Heterotopic gastric mucosa tissue within the Meckel’s diverticulum (MD) (hematoxylin and eosin staining, ×10). **(B)** Heterotopic jejunal mucosa tissue within the MD (hematoxylin and eosin staining, ×10).

A 5-year-old boy presented with a 4-month history of recurrent, intermittent abdominal pain accompanied by diarrhea. During this period, the child was evaluated at four hospitals without receiving a definitive diagnosis, and was then referred to our hospital. Physical examination on admission revealed no fever, a soft and flat abdomen without tenderness or rebound pain, and normal bowel sounds. Laboratory tests showed: white blood cell count 6.68 × 10^9^/L, hemoglobin 143 g/L, neutrophil percentage 49.70%, lymphocyte percentage 33.80%, platelet count 283 × 10^9^/L, and C-reactive protein 2.55 mg/L. Contrast-enhanced abdominal computed tomography (CT) revealed no significant abnormalities. Subsequently, intestinal color Doppler ultrasonography was performed. HFUS revealed on the longitudinal section a “glove-finger”-shaped hypoechoic structure protruding into the intestinal lumen within the ileum in the lower abdomen. One end was blind, and the other was connected to the adjacent intestinal wall, measuring approximately 65 mm in length and 7 mm in diameter. Within this “glove-finger” structure, hyperechoic mesentery and tubular anechoic mesenteric vessels were observed extending outward and continuing with the neighboring mesentery. The transverse section displayed a “target sign.” The outermost layer of the “target sign” corresponded to the intestinal wall; the middle layer consisted of an intestinal wall-like structure, no “reflex fold” was observed; and its center appeared hyperechoic (Figure 1). The ultrasound diagnosis was complete MD inversion. After preoperative preparation, the patient underwent transumbilical single-port laparoscopic partial small bowel resection and anastomosis. Intraoperative findings confirmed MD inversion (Figure 2). The patient was discharged 7 days after the operation with stable condition. The pathology of the specimen was consistent with MD. Both heterotopic gastric mucosa and heterotopic jejunal mucosa were present within the diverticulum (Figure 3). A 6-month follow-up revealed no discomfort or abnormalities in bowel movements.

## Discussion

### Overview of MD

Most MD cases remain asymptomatic throughout life and are discovered incidentally during imaging or autopsy. A minority of patients develop symptoms due to complications, with children having a higher likelihood of complications than adults (5, 6). The lifetime risk of complications is estimated at 4–6%, including gastrointestinal bleeding (31%), inflammation (25%), intestinal obstruction (16%), intussusception (11%), and internal hernia (11%) (6). MD is a true diverticulum, containing all layers of the intestinal wall. It frequently harbors heterotopic gastrointestinal tissue, most commonly gastric tissue, particularly in symptomatic cases (7). The resected specimen revealed the presence of both heterotopic gastric mucosa and heterotopic jejunal mucosa within the diverticulum.

### Etiology of MD inversion

Inverted MD, also known as intussuscepted MD, is a rare morphological variant and an uncommon cause of small bowel obstruction (SBO) (8). Some scholars describe the clinical progression of MD inversion in four stages: MD intussusception, partial MD inversion, complete MD inversion, and inverted MD secondary to intussusception (9).

The mechanism of inversion remains unclear but may involve factors such as heterotopic tissue, abnormal diverticular peristalsis, and the mobility of the diverticular tip (10). However, the association between heterotopia and MD inversion is inconsistent in the literature. Rashid et al. (2) found no heterotopic tissue in 41% of patients with inverted MD, whereas Xu et al. (11) reported heterotopia in all seven cases in their series. Negrea et al. (12) found a higher density of nerve fibers in the MD wall compared to the adjacent ileum, potentially promoting increased peristalsis and predisposing to inversion. Age-related decreases in intestinal nerve fiber density may explain the higher incidence of inversion in children. Additionally, lesions within the MD, especially space-occupying lesions such as lipomas, may increase the risk of inversion. Lovenish (13) reported a case in a 30-year-old woman in whom a lipoma at the diverticular tip caused inversion and intestinal obstruction.

### Clinical presentation of inverted MD

Because inversion of a Meckel’s diverticulum into the ileal lumen typically results in intestinal obstruction with symptoms including abdominal pain, hematochezia, nausea, vomiting, and altered defecation (14), cases of pure inversion without obstruction are exceptionally uncommon. In the report by Xu et al. (11), four of seven children with inverted MD had intestinal obstruction, all with 2–5 prior episodes of recurrent intussusception.

Although cases of pure inversion are exceedingly rare, they do occasionally occur, as exemplified by the present case which involved pure MD inversion without secondary intussusception or obstruction, presenting with non-specific initial symptoms of abdominal pain and diarrhea. The inversion of a Meckel’s diverticulum into the ileal lumen induces gastrointestinal motility disturbances, compromised intestinal blood flow, and ulceration, which may manifest as gastrointestinal bleeding and altered bowel habits. Nevertheless, due to its non-specific clinical presentation, pure inverted MD is frequently difficult to diagnose and is recognized as a major contributor to occult lower gastrointestinal bleeding (2).

### Preoperative diagnostic methods for inverted MD

Preoperative diagnosis of pure MD inversion is extremely challenging. Commonly used imaging methods include 99mTc-pertechnetate scintigraphy, enteroscopy or capsule endoscopy, CT, and ultrasonography. 99mTc scintigraphy has high specificity for MD but is susceptible to false negatives, requires strict examination conditions, and is not widely available (15). Capsule endoscopy can assist in MD diagnosis but requires high video quality (16), has limitations in precise lesion localization, and carries an increased risk of obstruction (17), warranting cautious use. CT is considered highly informative but often fails to identify MD in the absence of complications such as obstruction (8) and involves radiation exposure. In this case, contrast-enhanced CT did not yield a definitive diagnosis of MD inversion.

HFUS offers advantages in diagnosing MD in children. The ultrasonographic appearance of MD varies depending on its pathology and complications. Typically, an uncomplicated MD may appear sac-like or glove-finger-shaped, with one end communicating with the ileum, and the other end is blind. The wall is thick with distinct layers. Linear hyperechoic heterotopic tissue may be present. The lumen is usually anechoic and communicates with the adjacent bowel. When communication is wide, low intraluminal tension may cause diverticular collapse. When MD induces intussusception, it is typically at the leading point but often compressed by intussuscepted bowel or overlooked, leading to a missed diagnosis. In pure MD inversion, ultrasound reveals an abnormal structure protruding into the intestinal lumen, presenting as a “glove-finger sign” on the longitudinal view and a “target sign” on the transverse view, with central hyperechoic mesentery and anechoic tubular mesenteric vessels. In this case, these typical findings enabled accurate preoperative diagnosis.

### Differential diagnosis

The main differential diagnoses for inverted MD on ultrasound include inverted appendix, intestinal lipoma or lipoblastoma, and intestinal polyps. Duan et al. reported that the inverted appendix closely resembles inverted MD. The key distinguishing feature is location: the appendix inverts into the cecum, whereas the MD inverts into the ileum. The ileocecal valve serves as an important anatomical landmark (18). Inverted MD may sometimes be mistaken for a lipoma. Intestinal lipoma or lipoblastoma is relatively rare and appears as a hyperechoic mass protruding into the intestinal lumen, resembling the central hyperechoic mesentery in inverted MD. However, lipomas or lipoblastomas typically present as a mass without a surrounding hypoechoic intestinal wall-like structure. In inverted MD, the central hyperechoic mesentery typically appears band-like and is surrounded by the hypoechoic diverticular wall. On ultrasound, intestinal polyps usually appear as hypoechoic masses within the intestinal lumen with a stalk attached to the bowel wall, closely resembling inverted MD; thus, misdiagnosis is possible (19). However, colon polyps are more common; small intestinal polyps are less frequent, and polyps show homogeneous echogenicity or small cystic anechoic areas. MD is located in the ileum and exhibits a layered intestinal wall-like structure.

### Management

Surgical resection is the treatment of choice when MD causes complications. However, management of incidentally discovered asymptomatic MD during surgery for other reasons remains controversial, particularly whether to perform prophylactic resection (20). One study found a 5.3% risk of surgical complications following prophylactic resection (7). Some scholars thus argue that because MD has a low probability of causing symptoms, prophylactic resection increases postoperative risk with limited patient benefit (21).

Conversely, others contend that although the lifetime risk of MD complications is low, it does not decrease with age; therefore, they advocate resection when incidentally discovered (22). Some authors suggest that while Meckel’s diverticulum (MD) itself is a common congenital finding, its inversion is exceedingly rare. When it does occur, surgical removal is generally advised due to the associated risk of subsequent intussusception (14). In a study by Burjonrappa and Khaing (23), ectopic tissue was identified as the primary determinant for surgical intervention in patients with Meckel’s diverticulum.

While debate continues, surgical decisions ultimately depend on patient-specific factors and clinical judgment. In this case, because the child experienced recurrent abdominal pain and diarrhea, and given his overall good condition, we chose transumbilical single-port laparoscopic partial small bowel resection and anastomosis. Postoperative recovery was uneventful and complication-free.

## Conclusion

This case report describes an exceptionally rare and preoperatively challenging pediatric condition—pure inversion of Meckel’s diverticulum without obstruction. Its clinical presentation is non-specific and easily confused with other abdominal pathologies. The diagnostic process highlights the clinical value of HFUS as a first-line imaging modality for MD. Its advantages, real-time imaging, high resolution, and absence of radiation, allow sensitive detection of characteristic sonographic features of inverted MD within the intestinal lumen, providing decisive evidence for non-invasive, accurate preoperative diagnosis. For children with recurrent abdominal pain, clinicians and sonographers should maintain a high index of suspicion for this condition. Awareness of this rare entity, along with its characteristic sonographic features, may aid clinicians and sonographers in reaching an accurate preoperative diagnosis when encountering similar cases of unexplained abdominal pain in children.

## Data Availability

The original contributions presented in the study are included in the article/Supplementary material, further inquiries can be directed to the corresponding authors.
